# Exploring Work-Related Causal Attributions of Common Mental Disorders

**DOI:** 10.1007/s10926-014-9556-z

**Published:** 2014-12-03

**Authors:** Ingrid Blø Olsen, Simon Øverland, Silje Endresen Reme, Camilla Løvvik

**Affiliations:** 1 Uni Research Health, Uni Research, POB 7810, 5020 Bergen, Norway; 2 Department of Public Health, Norwegian Institute of Public Health, Oslo, Norway; 3 Department of Psychosocial Science, University of Bergen, Bergen, Norway

**Keywords:** Occupational health, Mental disorders, Return to work (RTW)

## Abstract

*Purpose* Common mental disorders (CMDs) are major causes of sickness absence and disability. Prevention requires knowledge of how individuals perceive causal mechanisms, and in this study we sought to examine work-related factors as causal attribution of CMDs. *Methods* A trial sample of n = 1,193, recruited because they struggled with work participation due to CMDs, answered an open-ended questionnaire item about what they believed were the most important causes of their CMDs. The population included participants at risk of sickness absence, and participants with reduced work participation due to sickness absence, disability or unemployment. We used thematic content analysis and categorized responses from 487 participants who reported work-related factors as causal attributions of their CMDs. Gender differences in work-related causal attributions were also examined. *Results* The participants attributed their CMDs to the following work-related factors; work stress, leadership, reduced work participation, job dissatisfaction, work conflict, social work environment, job insecurity and change, workplace bullying, and physical strain. Women tended to attribute CMDs to social factors at work. *Conclusion* Findings from this study suggest several work-related risk factors for CMDs. Both factors at the workplace, and reduced work participation, were perceived by study participants as contributing causes of CMDs. Thus, there is a need to promote work participation whilst at the same time targeting aversive workplace factors. Further, our findings indicate that work-related factors may affect women and men differently. This illustrates that the association between work participation and CMDs is complex, and needs to be explored further.

## Introduction

Although work participation is generally regarded as beneficial for mental health [1], there is ample evidence that workplace factors can influence mental health negatively and possibly lead to Common mental disorders (CMDs) [2–5]. Various workplace factors like long work hours [6], adverse psychosocial working conditions [7], and job insecurity [8] are all considered potentially harmful for psychological wellbeing [1]. Influential theoretical models in this area are the demand-control model [9] and the effort-reward imbalance model [5], which both focus on work stress derived by factors at the workplace. These models imply that high demands from superiors [9], low levels of subjective control [9] and lack of sufficient reward from superiors [5] cause work stress and mental strain.

The concept of illness perceptions can be applied to shed further light on the association between workplace factors and CMDs. *Illness perceptions* are mental models that include information about the following components; illness *identity*; its label and associated symptoms, its *timeline* or expected duration, its perceived *controllability*, its expected *consequences*, and the perceived *causes* of the illness [10]. According to the illness perception model, the individual utilizes information from the various components to cope with health-threatening stimuli and the resulting illness [10]. The various illness perception components have shown to predict patient outcomes within a range of somatic and mental conditions [11–15]. Further, illness perceptions are associated with sickness absence [16], and predict return-to-work (RTW) in somatic conditions [17–19], subjective health complaints [19, 20] and CMDs [20, 21]. The causal attribution component of illness perceptions is thought to influence various *health* *behaviors*, the kind of strategies people use to control and cope with their illness [22–25]. Recent findings show that people suffering from CMDs frequently attribute their CMDs to work-related factors [26]. Further, attributing illness to workplace factors may lead to sick listing as a form of palliative coping, which allows employees to escape workplace stimuli that are perceived as harmful [22, 23].

Today CMDs account for a larger proportion of the sickness absence load than any other disorders in Western countries [27–29]. It seems plausible that sick listed individuals who attribute CMDs to work-related factors may develop reluctance toward returning to work altogether in order to avoid these factors. Attributing CMDs to work-related factors may thus have implications for the occurrence and duration of sickness absence in this patient group. Further, causal attributions of CMDs to work-related factors may reflect risk factors for the development and/or maintenance of such disorders at the workplace. Finally, reduced work participation is also associated with CMDs [2–5]. Therefore, in this study we sought to examine work-related factors as causal attributions of CMDs.

## Materials and Methods

### Study Design and Procedure

Data analyzed in this study were collected as part of the “At Work and Coping” (AWaC) trial, a multicenter randomized controlled trial aimed at evaluating the effect of short-term work-focused cognitive behavior therapy (CBT) [30], and an adaptation of individual placement and support (IPS) [31] on RTW in CMDs (Trial registration—http://www.clinicaltrials.gov, with registration number NCT01146730). A detailed figure illustrating participant flow has previously been published elsewhere [26].

Information about the AWaC trial was distributed through local national insurance offices, other work rehabilitation services, general practitioners (GPs) and through the web. Participants were recruited by self-referral, referral from GPs, and through case managers at local national insurance offices or other vocational rehabilitation services. Participants were randomly assigned to a trial group that received short-term work-focused CBT and IPS, or a control group receiving usual care from Norwegian Labor and Welfare Administration (NLWA) services and GPs. Nine participants withdrew their consent after inclusion. Before inclusion all participants went through a brief assessment procedure lasting approximately 30 min. They were given detailed information about the study both verbally and in written form, with emphasis on participants’ right to withdraw from the study at any time without any explanation. Potential participants were assessed according to inclusion and exclusion criteria, and those eligible and willing were included. Prior to randomization, all participants completed baseline questionnaires involving data on demographic and background variables, physical and mental health problems, work participation and illness perceptions. Participants randomly assigned to the trial group (n = 629) began work-focused CBT after approximately 2 weeks. To promote usual care for the control group (n = 564), letters informing about group allocation were sent to the participants’ local national insurance offices or GPs. Follow-up questionnaires were administered by mail 6 and 12 months after inclusion, and registry data regarding work participation (sickness absence and long-term benefits) were collected from NLWA. Data used in this study are from baseline questionnaires. Questionnaire responses were registered in SPSS software, and text responses were transferred verbatim.

### Questionnaires

Causal attribution of CMDs was measured through the open-ended item of the Brief Illness Perception Questionnaire (B-IPQ) [32] included in the baseline questionnaire package. The B-IPQ assesses the different components of illness perceptions with nine single-item scales [33]. Further, the B-IPQ has shown to provide a rapid assessment of illness perceptions in ill populations and large-scale studies [32]. The open-ended item of the B-IPQ covers the causal component of illness perception, and has the following wording: “Please list in rank-order the three most important factors that you believe caused your illness”. Thus, each participant could report a maximum of three illness attributions. Clinical characteristics were measured using the Hospital Anxiety and Depression Scale (HADS) [33].

### Study Population

The AWaC trial included 1,193 participants from six different regions in Norway. All participants met specific predefined inclusion criteria; age between 18 and 60, self-reporting CMDs as obstructing work participation, RTW motivation, no ongoing psychiatric treatment elsewhere, and no severe mental illness, suicide risk, ongoing substance abuse or pregnancy. Participants could be actively working but at risk of sickness absence (n = 334), sick listed (n = 529) or receiving long-term benefits (n = 330) in the form of work clarification allowances or unemployment benefits. Thus, the study population varied with regards to work participation, ranging from actively working to full unemployment. For the current study, we explored responses from a subsample (n = 487) of the AWaC population. The subsample consisted of all participants who reported work-related factors when asked to present what they believe caused their CMDs.

### Ethical Considerations

The AWaC study was approved by Regional Committees for Medical and Health Research Ethics (REK vest). All principles in the Helsinki declaration were followed.

### Thematic Data Analysis

**Fig. 1:**
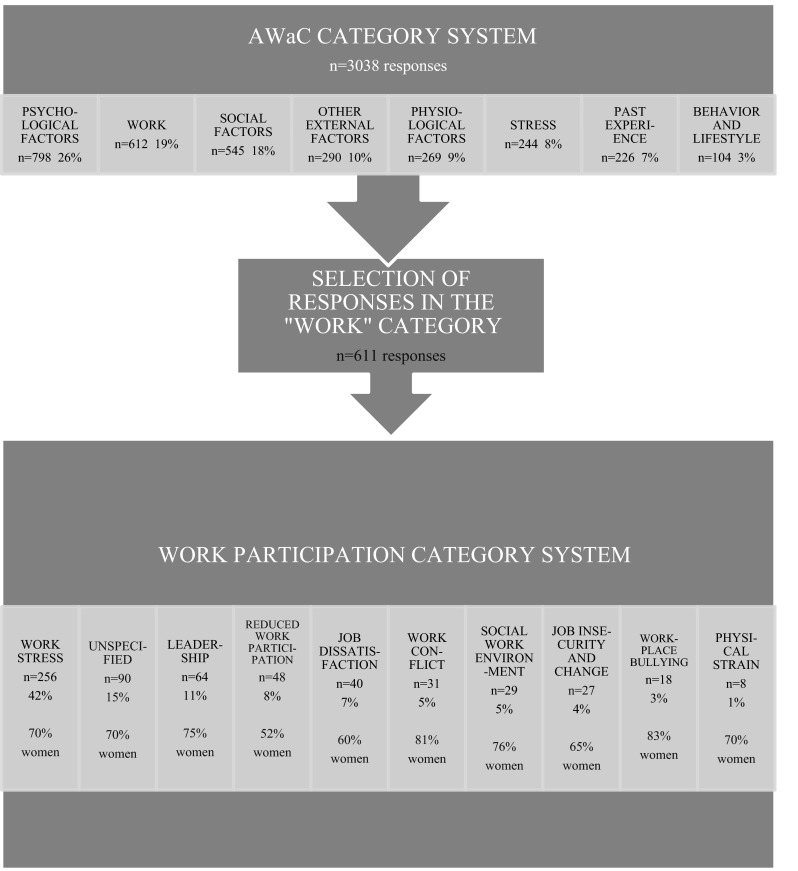
Flowchart of the thematic categorization process

Data used in this study are based on a thematic categorization of causal attributions from the AWaC population that was performed by the first author in a previous study [26]. The causal attribution categories are illustrated in the upper section of Fig. 1. For the current study, all responses reflecting work-related factors as causal attributions of CMDs were submitted to further analysis by the first author. Individual responses were sorted to identify different categories of work-related causal attributions. Analysis was done using a bottom-up, inductive procedure where category development was closely tied to and guided by data [34]. This entails that categories were added to the category system gradually as the dataset was investigated. When all responses had been categorized, they were assigned a primary code corresponding to their best-fitting category. This allowed for assessment of frequency distribution. Figure 1 illustrates the thematic categorization process.

### Inter-rater Reliability Assessment

To ensure the reliability of our analysis, inter-rater reliability was assessed for both the AWaC category system from which our responses were selected, and the work-related category system from the current study. The inter-rater procedure was performed by two individual inter-raters. To aid inter-rater coding, coding manuals that included category definitions, interpretations and inclusion criteria were written for both category systems (see Appendices 1, 2). One inter-rater was assigned to each category system. All responses in both data sets were coded based on the coding manuals. Prior to coding, inter-raters were allowed to discuss with the first author any questions they had regarding the categories and the manuals. There was no such discussion during inter-rater coding.

### Statistical Procedures

Descriptive statistics including frequency distributions were used to assess the distribution of all causal attributions for our study population, and then repeated for the work-related causal attributions. Gender-specific frequency distributions were calculated within each work-related category. All frequency distributions were computed based on primary codes. Cohen’s kappa, a numerical indication of the agreement between two raters of a categorical system [35], was used to assess inter-rater reliability of coding systems from both analyses.

## Results

Demographic and clinical characteristics of the entire AWaC population and our subsample are presented in Table 1. The AWaC population was characterized by a mean age of 40.2, a majority of women, education at college or university level and white-collar jobs. Compared to the total AWaC population, the subsample included more women, higher education level, more white-collar employees and somewhat higher total scores on clinical characteristics.

**Table 1:** Demographic and clinical characteristics of the AWaC population (n = 1,193) and subsample (n = 487)

Continuous variables	Population		Subsample	
	Mean	SD	Mean	SD
Age	40.2	9.6	40.7	9.36
HADS, total score	15.29	7.76	18.40	7.08

Categorical variables	N	%	N	%
Gender				
Female	800	67.1	335	68.8
Self-reported job status				
Actively working	334	28.0	120	24.6
Sick listed	529	44.3	262	53.8
Long-term benefits	330	27.7	105	21.6
Education				
University/postgraduate college	657	55.2	310	63.8
Occupation				
White collar	763	66.1	363	75.2
Mental health status (cut off ≥ 8*)				
Anxiety	926	78.2	362	74.8
Depression	633	53.5	274	56.6

* HADS score of 8 or above indicates symptoms in the clinical range

### Inter-rater Reliability

Inter-rater reliability as measured by Cohen’s kappa was high for both the category system for the entire AWaC population (K = 0.802) and the work-related coding system for the current study (K = 0.835).

### Work-Related Causal Attributions

The top section of Fig. 1 summarizes the categorization of causal attributions from the total AWaC population, performed in a previous study [26]. The most frequent causal attribution categories were Psychological factors, which included 798 responses (26 %), Work, which included 611 responses (19 %), and Social factors, which included 545 responses (18 %). Findings from the current study regarding categories of work-related causal attributions are summarized in the bottom section of Fig. 1. The categories identified were as follows: Work stress, Leadership, Reduced work participation, Job dissatisfaction, Work conflict, Social work environment, Job insecurity and change, Workplace bullying, Physical strain and Unspecified. The most frequent category was Work stress, covering 256 responses (49 %). Gender distributions within each category are reported in Fig. 1. The following categories were identified:

#### Work Stress

This category was interpreted as encompassing causal attributions to the psychological experience of work stress, mental workload, high demands at work and work-related burnout. In addition, the work stress category was of significant interest as it was assumed to potentially capture the psychological toll of Western work culture. Descriptive of this category were responses such as “extensive workload”, “too much work” “stress at work”, “too many work assignments”, and “burn-out, too extensive workload for too long”. It seems that participants in this category struggled with extensive workloads, multitasking, and the subjective feeling of stress or burnout related to work. Many participants also referred to stress caused by the double burden of work and family—“too much to do at work and at home”—indicative of the inability to combine family and professional roles.

#### Leadership

This category was constructed to capture causal attribution of CMDs to negative experiences related to superiors in a workplace hierarchy. Examples of responses were “the leader at work”, “diffusion of responsibility by management”, “my relationship with my boss”, and “lack of guidance at work”. Participants described perceived lack of support and understanding from workplace management, lack of training, guidance and individual arrangements in relation to work tasks, conflicts with management, diffusion of responsibility and lack of management skills, and perceived conflict within the management group.

#### Reduced Work Participation

This category was interpreted as covering causal attribution of CMDs to unemployment, sickness absence and disability. Examples of responses placed in this category were “labeled as incompetent, and shut out from working life”, “think a lot about my sickness absence”, “long-term unemployment”, “lost job, economic problems”, “cut-backs at work – no job”, “can’t find work even though I do a lot of applying”, “unemployment worsened my situation”, “loss of job/steady income/work identity”, “unfair firing”, and “fired from work after 27 years”. These responses reflect both reduced work participation in itself, and the psychological impact of reduced work participation.

#### Job Dissatisfaction

This category was created to cover causal attribution of CMDs to job dissatisfaction for reasons that were not covered by other categories. Examples of responses were “career choice”, “wrong kind of work”, “I didn’t get the job I wanted”, and “stagnation at work, need for change”. The Job dissatisfaction category thus captured dissatisfaction with fairly global work factors related to job type.

#### Work Conflict

This was the most prominent of the psychosocial categories, and was developed to encompass responses regarding conflict at the workplace. Participants reported “work conflict”, “conflicts with pupils”, “conflict with parents in work situation”, “conflicts with customers at work” and “problems with aggressive parents at work”. This reflects diverse forms of work-related conflicts with colleagues, customers and people who are indirectly affiliated with the participants’ work.

#### Social Work Environment

This category was constructed to cover negative social environment at work in the form of negative or lack of collegial relationships, and lack of social support from colleagues. Participants referred to “relations to work colleagues”, “frustration at workplace”, “lack of understanding and respect from colleagues”, “isolated work situation”, “lack of teamwork”, “bad climate at work”, and “too few colleagues in my work environment”. Responses reflected lack of support from colleagues, having too few colleagues, negative work climates, problems with romantic relationships at work, and difficult relationships in general with colleagues.

#### Job Insecurity and Change

This category was interpreted as pertaining to attribution of illness to various forms of instability and change at the workplace. It captured both unpredictability regarding work assignments and job descriptions, and insecurity regarding future employment. Participants reported “uncertainty regarding work situation”, “afraid to call in sick”, “unpredictable work day”, “uncertainty regarding future and work”, “new job”, “new tasks”, “unclear work instructions”, and “reorganization at work, lasting for 2 years”. Responses reflected concerns about new job assignments, changing routines, new colleagues, starting new jobs, fear of losing ones’ job, and organizational changes at work.

#### Workplace Bullying

This category was created to encompass responses regarding all forms of bullying and harassment at the workplace. Examples of response items were “subjected to psychological violence at home and at work”, “harassment case at work”, “several episodes of violence at work”, “harassed by my superior”, “sexual harassment at work” and “threats from work colleague”. Responses reported various forms of workplace bullying, including violence, sexual harassment and threats from management, colleagues, customers, pupils and other people participants encounter at work.

#### Physical Strain

This category was developed to capture all forms of exposure to physical strain and harm at work. Participants reported “work injury”, “work accident”, “nursing job, is exposed to heavy lifting”, “I became really sick at work”, and “heavy manual labor”. This category reflected work-related physical strain that included injuries, accidents, illness, and heavy physical workloads.

#### Unspecified

This category was constructed to include all work-related responses that did not offer further specification of work-related factors as causes of CMDs, and, thus, could not load on any of the other categories. Participants typically reported “work”, “my job” and “factors at work”.

## Discussion

The current findings indicate that people struggling with work participation due to CMDs frequently perceive their CMDs as caused by work-related factors. This study identified a range of such work-related factors: Workplace leadership, job dissatisfaction, job insecurity and organizational changes, physical strain and social stressors like workplace conflicts and bullying, all associated with CMDs [1, 36–45]. Further, our findings indicate that some participants attribute CMDs to reduced work participation in the form of sickness absence, disability and unemployment. These findings highlight the complex relationship between work participation and CMDs. Several aspects of working life are perceived as detrimental to mental health, but so, too, is not being able to work. Finally, our findings revealed gender differences with regards to causal attributional style. Women tended to attribute CMDs to social factors at work in the form of bullying, conflict and leadership. Men, on the other hand, made more attributions of CMDs to reduced work participation, job insecurity and job dissatisfaction. Previous findings suggest that white-collar women tend to employ social support coping [46]. Thus, one may hypothesize that the gender differences in attributional style is caused by gender differences in work coping style.

### Strengths and Limitations

An important strength of this study is the size of the AWaC population, and the subsample examined in the current study. This enhances the generalizability of the current findings, and may point to workplace risk factors for employees with reduced work participation due to CMDs. Participants had highly varying degrees of work participation at time of inclusion. Some participants were sick listed while others at risk of sickness absence, and some were receiving long-term benefits. This variation in work participation reflects the Norwegian working age population in general. In addition, participants were referred from several different agents. This adds to the generalizability of the findings, as it enhances study population heterogeneity. The female dominance in our subsample is also a reflection of society in general, as the majority of people suffering from CMDs are female [47]. A consequence of this, however, is that our findings may not be generalizable to the male population. Further, the distribution of blue- versus white-collar workers in the subsample also limits the generalizability of our findings. As the subsample has a higher education level and consists of 75 % white-collar workers, the findings may be generalizable to white-collar populations only.

An additional strength is the use of self-report data. The data consists of participants’ own quotes, and thus presents participants’ own experiences. Further, in the current study a large patient group is permitted to voice their concerns, and point out possible deleterious contextual factors in their work environment. This may in turn inform the design of future RTW-interventions. However, the use of an open-ended question entails that responses vary substantially with regards to content; some quotes are long and quite specific, others are short and points to work in general. This is evident in the “Unspecified” category from our findings.

Further, a central characteristic of the method used in this study is the fusion of data and the authors’ interpretations and construction of meaning [35]. Our interpretation of text responses may not adequately have captured the participants’ intentions and views. This is an inherent limitation to qualitative methodology, but also recognized as one of its strengths [34].

Finally, a comment has to be made with regards to the low frequency of the categories Work conflict, Workplace bullying and Social work environment. The categories reflect work-related factors with a known association with CMDs [1], and thus, one should expect larger frequencies of these categories. The reason for the low frequencies may be the participant recruitment procedure in the AWaC. Candidates had to report CMDs as obstructing work participation in order to be included in the study population. Thus, candidates reporting other factors, for example work conflicts, as obstructing work participation, may have been excluded from study participation.

### Implications from the Current Findings

The fact that a large majority of our subsample attributed their CMDs to factors at the workplace is important, as we currently see an increase in the perceived exposure to work stress among employees in several European countries [28]. As causal attributions elicit emotional reactions [25], one can hypothesize that attributing CMDs to work-related factors leads to negative perceptions of and feelings toward work. This may in turn foster reluctance toward returning to work because the individual fears a relapse in CMDs. Thus, the large majority of our subsample that attribute CMDs to factors at the workplace may be at risk of prolonged sickness absence spells. This may in turn be detrimental due to the known association between reduced work participation and CMDs—an association that also is implicated by the “Reduced work participation” category among our findings.

Further, the current findings point to possible risk factors for CMDs at the workplace. The work-related factors identified in this study are diverse and cover many aspects of working life, including psychosocial factors, workplace leadership, organizational changes and the effects of reduced work participation. This illustrates the complex association between work participation and mental health; several factors at the workplace are perceived as causing CMDs, but so, too, is reduced work participation.

In addition, a point needs to be made with regards to the “Work stress” category, which was the most frequent work-related causal attribution in this study. Measuring work stress is complicated, as work stress is subjectively perceived rather than objectively defined [1]. Some have pointed to a discrepancy between subjective and objective measures of work stress [48, 49]. Participants’ causal attributions may be influenced by cultural trends and dominating common-sense explanations communicated through public media channels [25]. An example is the common trend in Western countries towards attributing CMDs to the stress of modern life [25]. Thus, causal attribution of CMDs to work stress may be the result of cultural influence on participants’ illness attributions. It has also been hypothesized that work stress is derived from within the employee, and may be created through employees’ active use of coping strategies at work [48]. An example is the activity of *job crafting*, which refers to the various actions employees take to shape and redefine their jobs [50]. Job crafting is done by changing three work-related factors; work tasks, the cognitive task boundaries of a job, and the amount and quality of social interaction at work [50]. The job crafting framework is based on the assumption that employees control their working situation and its associated responsibility to a large degree. The claim that work stress is internal, that is, created by employees themselves through job crafting, calls into question the basis of work stress theories like the Demand-Control model and the Effort-Reward Imbalance model. The internal perspective also adds to our understanding of the complex nature of work stress, and the active part employees may be playing.

A considerable number of the participants in this study referred to the fairly general concept of work stress. The lack of details in participants’ responses illustrates the need to further explore causal attributions of CMDs to work-related factors using other methodological approaches. To exemplify, longitudinal studies can be applied to investigate whether causal attribution of CMDs to workplace factors predicts future sickness absence or long-term benefits like disability pension. Qualitative studies should be designed to extract information about specific workplace factors, rather than capturing the general concept of work stress. Semi-structured interviews and focus group studies may shed light on how and why various workplace factors are perceived as contributing to or maintaining CMDs among employees. Gender differences could also be investigated further in these settings to explore differences in attributional styles, and whether men and women need different workplace interventions in order to enhance work participation. Self-reported effects of downsizing, job insecurity and changing work descriptions could be explored by selecting participants working for corporations undergoing organizational changes. Results from the forementioned studies could potentially shed light on the complex association between work participation and CMDs.

## Conclusion

The current study explored work-related factors as causal attributions of CMDs. The following work-related factors were identified; work stress, leadership, reduced work participation, job dissatisfaction, job insecurity and change, work conflict, social work environment, workplace bullying and physical strain. The current findings point to several work-related risk factors for development and maintenance of CMDs. An important implication is that both factors at the workplace *and* reduced work participation are perceived as causing CMDs. Thus, in order to prevent CMDs, our findings indicate the importance of maintaining work participation, whilst at the same time targeting aversive workplace factors. Further, our findings indicate that women and men tend to attribute CMDs to different work-related factors. This may entail that work-related factors affect women and men differently. Thus, our findings illustrate that the association between CMDs and work participation is complex, and needs to be explored further in other settings and populations, and with other study designs.

## Appendix 1: Coding Manual for the AWaC Category System

Categories from the preliminary analysis are work, stress, heredity, personal relationships, bullying, childhood, negative life events, somatic diagnoses, somatic complaints, injuries and accidents, pregnancy and childbirth, economy, death of significant other, lack of social support, life situation, unpredictability, sexual orientation, maltreatment, other external factors, personal expectations, self-regulation, self-image, psychiatric diagnoses, psychological complaints, emotional reactions, lack of coping, personal vulnerability, behavior and lifestyle, responsibility and do not know.

All items are to be given at least one code, referred to as the primary code. When a response item fits several categories, it is given additional codes corresponding to all relevant categories. The primary code is based on the first-mentioned category in the response item, or the code considered to be best-fitting. To exemplify, the item “work and family” is primarily coded in the Work category, and given an additional code for the Personal relationships category. Descriptions with inclusion and exclusion criteria for the categories are as follow:

### Work

This category includes all items that are work-related in some way, like workload, work stress, work satisfaction, psychosocial work environment, work hours, work conflicts and unemployment. Items that mention bullying at work are excluded, and placed in the Bullying category.

### Stress

This Category contains items that mention stress/strain/external pressure without any specific stressor (for example “stress”), or several stressors that interact to cause stress (e.g. “stress – work and domestic”). The key is that the person pictures stress as the causal factor. Items are also included if they contain words that describe a load that is too much to handle, for instance “too much to do at work and at home”. Items are not included if they

- Contain words that refer to psychological processes that cause stress, or lack of psychological capacity to cope with stress. These factors are regarded as internally controllable, and the items are to be placed in one of the internal categories.
- Contain several specific factors without relating them to stress (to exemplify, “work and family”. These items are to be coded like double-barreled items mentioned above).
- Attribute stress to one of the other more specific categories, for instance “marital stress” and “work stress”. These are placed in the category that match the stressor (in these cases, Personal Relationships or Work).

### Heredity

This category includes all items related to genetic dispositions, such as “heredity”, “heritability”, “it runs in the family”, “genes”. Items are excluded if they refer to

- Passing on in the family of factors that are not genetic, for instance “social heritage”.
- Disorders/diseases that are thought to be hereditary, but heredity is not mentioned specifically (such as “bipolar disorder”).

### Personal Relationships

This category contains responses that refer to significant others and close relationships; family relations and family roles, love and friendships, significant others failing to meet expectations in such relationships (for instance “betrayal”), and domestic factors, for example “domestic situation”. The person may or may not contribute to the problem. Items are excluded when they refer to

- Death of significant others, these are placed in the death of significant other category.
- Lack of social support in a wider sense, such as “lack of respect”, without mention of significant other. These are placed in the lack of social support category.
- Psychological reactions to such relationships, these are placed in one of the internal categories.
- Actions of significant others that are not relational (to illustrate, “my sons drug abuse”, “my father’s illness”). These are placed in the negative life events category.

### Bullying

Items are put here that mention bullying specifically, for example “bullying” or “harassment”. Items are also placed here even if

- The bullying is time-limited, and therefore could have been placed in the negative life events category (such as “was bullied in first grade”).
- The bullying happened in childhood, and therefore could have been placed in the childhood category.
- The bullying takes place at work, and could be placed in the work category.

### Childhood

This category pertains to items referring to childhood and experiences in childhood. If other categories also are mentioned, for instance “emotional abuse in childhood”, the item belongs to this category. The key is that the respondent traces the factor back to childhood. Items are also placed here if they not mention childhood specifically, but are clearly related to childhood, such as “absent father”. Items are excluded if they mention bullying.

### Negative Life Events

Items are placed here when they refer to traumatic experiences or negative events that are relatively limited in time, for example “rape”. Items are excluded if they refer to

- Relational difficulties or processes where the person also may contribute to the problem, for instance “marriage problems” or “conflict with boyfriend”, these are placed in the personal relationships category.
- Factors that are relatively situational, general or chronic, such as “unpredictable situation”. These items are placed in the other external factors category.
- Death of a significant other, these are placed in the death of significant other category.
- Bullying, these are placed in the bullying category.
- Accidents, these are placed in the injuries and accidents category.
- Divorce/break up, these are placed in the personal relationships category.

### Somatic Diagnoses

This category contains responses that refer to clinical somatic diagnoses, for example “cancer” and “migraine”. It also includes responses that refer to surgical treatment of such diseases. Items are excluded if they refer to

- Somatic symptoms or health complaints, for instance “headache” or “back pain”.
- Psychological reactions to such diseases, these are placed in one of the internal categories.

### Somatic Complaints

This category includes responses that refer to somatic symptoms and subjective health complaints that do not constitute a diagnosis, such as “back pain” and “head ache”. This includes complaints after surgical treatment, exemplified by “pain after cancer surgery”. Items are excluded if they refer to psychological diagnoses, complaints or symptoms, for example “fatigue”, “worrying” or “sleep difficulties”. These are placed in one of the internal categories.

### Injuries and Accidents

This contains responses that refer to somatic injuries or accidents, such as “car accident” or “arm fracture”, or surgical treatment that are unrelated to somatic diagnosis, e.g. “amputation”. Items are excluded if they refer to somatic diagnoses or psychological complaints or symptoms following such incidents, such as “back pain after car accident”. These are placed in one of the internal categories.

### Pregnancy and Childbirth

This category includes responses about pregnancy and childbirth.

### Economy

This category includes responses that refer to financial problems, for example “economy” or “debt”.

### Death of Significant Other

This category includes responses concerning death of significant others. Items are excluded if they refer to psychological reactions to such losses, these are placed in one of the internal categories.

### Lack of Social Support

Items are put here when they contain responses that describe other people’s lack of meeting the person’s social and emotional needs, for instance “lack of respect” or “violation of trust”. The category captures lack of support in the person’s social network. Items are excluded if significant others are mentioned, these are placed in the Personal relationships category.

### Life Situation

This category contains items referring to a difficult life situation. Items are excluded if they describe life situation more specifically, exemplified by “difficult life situation because of workload”. This is placed in one of the more specific external categories, in this case Work.

### Unpredictability

This category pertains to items regarding unpredictability as causal factor. Here we picture that the respondent perceives the external situation as unpredictable.

### Sexual Orientation

Items are placed here when they refer to sexual orientation, and problems dealing with this.

### Maltreatment

This category includes items that mention maltreatment by health professionals.

### Other External Factors

Items are placed here when they don’t fit in any of the more specific categories, but still refer to clearly external factors, e.g. “the ways of the world”, “school”, “environment”, “private things”. They are often general in character. Items that refer to psychological states are excluded, and placed in one of the internal categories.

### Expectations

Items are placed here when they refer to the respondent’s own expectations regarding own achievements, perfectionism, need for achievement, lack of and fear of not meeting these expectations, such as “expects too much”, “fear of not being good enough”. It is crucial that the expectation is the person’s own. Items are excluded if they refer to

- Other people’s expectations, these are placed in external category other external factors.
- Expectations of significant others, they are placed in the personal relationships category.

### Self-regulation

Items are placed here when they refer to internal difficulties regulating external stressors, or over-focusing on outer demands at the cost of own needs. To exemplify, “difficult to say no”.

### Self-image

Includes items that refer to self-image, self-esteem and self-worth, for instance “low self-esteem”, “lack of belief in myself”, “negative self-image”.

### Psychiatric Diagnoses

This includes items that refer to specific psychiatric diagnoses, such as “depression” and “PTSD”. Items are excluded if they refer to psychological complaints and symptoms, and somatic diagnoses.

### Psychological Complaints

This category contains items that refer to subjective health complaints and symptoms that are psychological of nature, for example “rumination” or “sleep disturbance”. Items are excluded if they refer to specific psychiatric diagnoses.

### Emotional Reactions

Items are put here if they refer to psychological reactions of emotional nature, exemplified by “grief”. Reactions that are less intense, and can be viewed as a personal tendency or personality trait, for instance “worrying” or “guilt”, are placed in the category for psychological complaints.

### Lack of Coping

This category is related to the classic definition of coping, where the person has sufficient resources to deal with external demands. It contains responses that describe maladaptive coping strategies, or lack of ability to cope, such as “lack of coping with divorce”, “lack of control” or “social isolation”.

### Personal Vulnerability

This category contains responses that refer to personality traits and tendencies that are viewed as fairly stable in psychological literature or by the respondent, for example “vulnerability”, “temperament” and “personality”. Items are excluded if they refer to tendencies that are symptoms of psychiatric diagnoses or psychological health complaints, exemplified by “worrying”. These are placed in the psychological complaints category.

### Behavior and Lifestyle

This category contains responses that refer to the person’s own actions or behavioral strategies, for instance “moved abroad” or “drug abuse”, and personal lifestyle, e.g. “diet”.

### Do Not Know

This category pertains only to responses reflecting that participants do not know which factors they believe caused their illness.

### Responsibility

This category contains items referring to responsibility as causal factor of disorder.

## Appendix 2: Coding Manual for the Work-Related Category System

Categories from the selective analysis are work stress, leadership, reduced work participation, job dissatisfaction, work conflict, social work environment, job insecurity and change, and physical strain. All items are to be given at least one code, the primary code. Response items that do not specify aspects of the work situation, like “work” or “bad work situation”, are to be coded in the “Unspecified” category. When a response item fits several categories, it is given additional codes corresponding to all relevant categories. The primary code is based on the first-mentioned category in the response item, or the code considered to be best-fitting. To exemplify, the item “workload and conflict at work” is primarily coded in the Work stress category, and given an additional code for the Work conflict category. Descriptions with inclusion and exclusion criteria for the various categories are as follow:

### Work Stress

This category includes all responses referring to excessive workloads, pressure and/or expectations from superiors, and lack of coping at work. Items are not put in this category if they mention social stressors; these are to be coded in one of the categories pertaining to social processes. Typically, responses are related to too extensive workloads or work hours, multitasking, the subjective feeling of stress or burnout related to work, and stress caused by the double-burden of work and family.

### Leadership

The category pertains to causal attribution of CMDs to negative experiences related to superiors in a workplace hierarchy. Responses may refer to lack of support and understanding from workplace management, lack of training, guidance and individual arrangements in relation to work tasks, conflicts with management, diffusion of responsibility and lack of management skills, conflict with leaders and perceived conflict within the management group and hostile or unfair bosses. Hostile leadership is included in this category because it is viewed as a different phenomenon than bullying: It is regarded as a general tendency in these bosses´ leadership styles, not directed at individual employees. Responses are excluded from this category and coded in the Workplace bullying category if they refer to leaders targeting individual employees negatively.

### Reduced Work Participation

Items are placed in this category that refer to reduced work participation in the form of sickness absence, disability and unemployment, and problems relating to this.

### Job Dissatisfaction

This category contains all items mentioning job satisfaction, without mentioning a reason for lack of job satisfaction that can be placed in any of the other categories. Responses typically refer to not getting the right kind of job, or not being satisfied with work tasks.

### Work Conflict

Items are placed in this category if referring to work conflicts with colleagues and customers, and conflicts where the other party is not specified. Responses referring to conflicts with superiors are to be coded in the category leadership.

### Social Work Environment

This category is thought to capture negative social climate and lack of social support at work. Respondents may refer to lack of support from colleagues, having too few colleagues, negative work climates, problems with romantic relationships at work, and difficult relationships in general with colleagues. This category also covers all forms of bullying and harassment at the workplace, and includes responses referring to bullying, violence, general harassment, sexual harassment and threats from management, colleagues and customers.

### Job Insecurity and Change

This category is thought to capture causal attribution of CMDs to different forms of job insecurity, unpredictability, job change and problems with dealing with a new job, fear of getting sick listed, reorganization and organizational changes at the workplace, and problems related to this.

### Workplace Bullying

This category covers all forms of bullying and harassment at the workplace, and includes responses referring to bullying, violence, general harassment, sexual harassment and threats from management, colleagues and customers.

### Physical Strain

This category pertains to all response items regarding work-related physical injury, accidents and bodily strain. Items are included that mention shiftwork, as this is thought to be straining primarily because of sleep deprivation or disrupted circadian rhythm.
